# A Travesty on Religion and Law

**Published:** 1894-02

**Authors:** 


### A TRAVESTY ON RELIGION AND LAW.

The negro rapist, Ed. Nichols, was executed at Austin, January 12, ult., under the most extraordinary circumstances. It was a human sacrifice with religious rites, conducted by the negroes upon the scaffold, the State's sheriff acting as High Priest.

The ostensible object of hanging is to lessen crime. It is held that a display of the law's power and majesty will awe the evil disposed and restrain them from the commission of crime. That the gallows,—nay, the *stake*, has no terror for the negro rape-fiend; that plain and swift hanging, nor burning at the stake will lessen the occurrence of rape has been abundantly proven in Texas, for it is notorious that the crime, and in its worst form, is on the increase, nothwithstanding the swift and terrible vengeance in most cases visited upon the offender. Capital punishment then, is useless, even in its most horrible form. What will be the effect when it is made fascinating, attractive,—really seductive, nay—even *glorious*, as in the case of Nichols?

It was well meant, no doubt;—a mistaken notion of charity on the part of the sheriff, to permit the friends of the wretch to hold religious services under the very shadow of the gallows, but it was a grave error of judgment. It was not only permitting religion to be made a farce; it was not only an affront to the community of Christian people, but by this act the execution lost its character as such, was shorn of its horrors, and lost all terror for the evil-doer, but it became an Africo-religious *rite* attending the sacrifice of a *hero*, a hand-made, or, as alleged, a prayer-made saint, and as such it became an example which appeals strongly to the superstitious nature of the negro, and invites to emulation. It is *dangerous* in the extreme, and our people should beware. The crown of martyrdom is thus offered as a premium on rape. The spectators were assured by the negro minister officiating that Nichols' soul had been "washed in the blood of the Lamb and made clean," and that he was "going straight to the bosom of God." "Nearer my God, to Thee"—that grand old hymn so dear to every Christian heart, was sung as an accompaniment to *fixing the noose and adjusting the black cap*,—and the ghoul-like spectators, perched like buzzards on adjacent roofs and walls, were requested to "join in." Desecration—profanation—*sacrilege* can go no further; religion is made a farce—and capital punishment a burlesque; worse than a burlesque, because, as stated, it becomes fascinating, and appeals to the brute nature strongly: it says, "go thou and do likewise, if you, too, would wear the crown of martyrdom and go to glory."

The JOURNAL enters its most earnest protest against a repetition of the disgraceful scene. Let the condemned have all the "benefit of clergy,"—all the comfort, solace and hope he can find in religion, prayer and song,—but in the name of all that is decent, let it be done in the cell, privately, and let not the awful majesty of the law, in the infliction of the death penalty, be travestied, and its purpose and object thwarted; nay, reversed, and *made to incite* to that which it is intended to prevent!

